# Spike specific IgG3 and nucleocapsid IgG response in serum serve as distinguishing immunological markers between SARS-CoV-2 infection and vaccination

**DOI:** 10.3389/fimmu.2025.1518915

**Published:** 2025-03-27

**Authors:** Marjahan Akhtar, Md. Rashedul Islam, Fatema Khaton, Fatima Rahman, Tausif Adnan Sami, Imam Tauheed, Tasnuva Ahmed, Afroza Akter, Ishtiakul Islam Khan, Zahid Hasan Khan, Prasanta Kumar Biswas, Edward T. Ryan, Sayera Banu, Tahmina Shirin, Fahima Chowdhury, Ashraful Islam Khan, Taufiqur Rahman Bhuiyan, Firdausi Qadri

**Affiliations:** ^1^ Infectious Diseases Division, International Centre for Diarrhoeal Disease Research Bangladesh (icddr,b), Dhaka, Bangladesh; ^2^ Division of Infectious Diseases, Massachusetts General Hospital, Boston, MA, United States; ^3^ Department of Medicine, Harvard Medical School, Boston, MA, United States; ^4^ Department of Immunology and Infectious Diseases, Harvard T.H. Chan School of Public Health, Boston, MA, United States; ^5^ Institute of Epidemiology, Disease Control and Research (IEDCR), Dhaka, Bangladesh

**Keywords:** COVID-19, vaccine, infection, IgG3, nucleocapsid

## Abstract

**Background:**

Both SARS-CoV-2 infection and COVID-19 vaccines elicit immunological responses. However, it is difficult to distinguish responses generated after vaccination versus natural infection.

**Methods:**

We investigated SARS-CoV-2 spike receptor-binding domain (RBD) and nucleocapsid-specific IgG and RBD specific IgG subclasses (IgG1, IgG2, IgG3 and IgG4) responses using ELISA in four different groups; (1) COVID-19 patients (n=39) with varying disease severity and (2) COVID-19 vaccinated individuals (n=24, both adenovirus/mRNA based) (3) vaccinated after infection (n=39) and (4) patients experienced breakthrough infection (n=14), in Bangladesh.

**Results:**

Both COVID-19 patients and vaccinees developed robust RBD-specific IgG responses. In contrast, nucleocapsid specific IgG responses were found in patients but not in vaccine recipients. A distinct IgG subclass antibody response was observed in COVID-19 patients compared to COVID-19-vaccinated individuals. Specifically, COVID-19 patients exhibited elevated levels of both IgG1 and IgG3, with IgG3 dominating in the early phase of infection (days 1-7) followed by a subsequent increase in IgG1. Conversely, COVID-19 vaccination predominantly induced IgG1 responses without a concurrent rise in IgG3. This effect was more evident when a significant rise of IgG1 but not IgG3 was observed in patients who received COVID-19 vaccines after 90 days of infection. However, following breakthrough infection, we observed an increase in both IgG1 and IgG3. All of these findings collectively indicate that COVID-19 vaccination predominantly induces IgG1, whereas natural infection can elicit responses in both IgG1 and IgG3 subclasses.

**Conclusion:**

The findings highlight RBD-specific IgG3 as well as nucleocapsid IgG as crucial markers for differentiating between vaccination and natural infection and suggest these assays have utility for longitudinal monitoring of vaccinations and for establishing SARS-CoV-2 correlates of protection.

## 1 Introduction

The recent pandemic of Coronavirus disease-19 (COVID-19), caused by severe acute respiratory syndrome coronavirus-2 (SARS-CoV-2), continues to pose a significant global health threat. Since its emergence in December 2019 in China, the pandemic has resulted in over 700 million confirmed cases and approximately 7 million deaths worldwide by April 2024 (1). The rapid development and deployment of COVID-19 vaccines have been played a vital role in the global efforts to curb virus-associated mortality. The pandemic has also prompted extensive research in understanding immune responses elicited by both SARS-CoV-2 infections and vaccination, which are critical in understanding the body’s defense mechanisms against the virus (2, 3). Both infection and vaccination trigger B and T cell mediated immune responses (4–7). The receptor-binding domain (RBD) of the spike S1 subunit has been a particularly important immunologic target because of its essential role in binding of the virus to the human angiotensin-converting enzyme 2 (ACE2) receptor, which facilitates SARS-CoV-2 entry into host cells (8–10). Natural infection with SARS-CoV-2 induces a broad immune response, targeting multiple viral proteins, including the spike (S) and nucleocapsid (N) protein. This comprehensive immune response has the potential to provide robust and long-lasting immunity, although its effectiveness and duration can vary among individuals (11, 12). Our previous studies on COVID-19 patients suggested that the magnitude and durability of antibody and memory B cell responses following SARS-CoV-2 infection depend on the severity of the disease (7, 12, 13). Patients with moderate to severe COVID-19 mounted significant levels of RBD-IgG antibodies for up to six months to one year, while asymptomatic or mild cases had detectable IgG antibodies for op to 3 months post-infection (7, 13). COVID-19 vaccines, especially messenger RNA (mRNA) vaccines such as BNT162b2 (Pfizer-BioNTech) and mRNA-1273 (Moderna) as well as adenovirus-based vaccines like ChAdOx1-S, have demonstrate robust IgG responses against RBD (14).

Differentiating between immune responses induced by infection and those elicited vaccination is a challenge due to the reliance on spike or RBD-based immunological responses, as well as variability in antibody levels. This overlap in immunological responses complicates determination of the origin of the response using standard serological assays. The N protein, which is not present in the approved COVID-19 vaccines, has been proposed as a potential marker to distinguish between vaccination-induced or infection-induced immunity (15).

However, distinguishing between the IgG subclass responses elicited by natural infection versus vaccination can provide valuable insights. For instance, individuals with a history of SARS-CoV-2 infection typically exhibit a broader spectrum of IgG subclasses, including IgG1 and IgG3. In contrast, SARS-CoV2 vaccination has been shown to predominantly induce IgG1 responses, with relatively poor induction of IgG3 (16). To explore whether serum IgG subclass antibodies could serve as distinguishing markers between infection and vaccination, this study investigated RBD-specific IgG subclass responses across four distinct groups: COVID-19 patients, vaccinated individuals (adenovirus/mRNA-based), individuals vaccinated post-infection, and patients who experienced breakthrough infections. The ability to distinguish between these responses based on specific IgG subclasses and their targets enhances our understanding of the immune landscape and is essential for effective public health interventions.

## 2 Materials and methods

### 2.1 Study participants

Participants enrolled in two of our previously conducted studies in Bangladesh of COVID-19 patients and vaccinees were included in this analysis. Naturally infected COVID-19 patients (n=39, RT-PCR test positive) with varying degrees of disease severity were enrolled between 2020-2021. Disease severity was based on clinical symptoms, such as fever, cough, oxygen saturation, breathlessness according to WHO guidelines. These participants received COVID-19 vaccines >90 days after infection. In addition, during the time of patient enrollment, healthy participants (n=20) with no history of COVID-19 symptoms and RT-PCR tested negative were enrolled. We also studied COVID-19 vaccinees (n=28) who received two doses of COVID-19 vaccine- either the adenovirus vector based ChAdOx1 nCoV-19 (Covishield, Serum Institute of India) or mRNA-1273 (Moderna) or BNT162b2 (Pfizer-BioNTech) and had no history of prior SARS-CoV-2 infection. Before enrollment and during subsequent visits, participants underwent interviews where information about their age, gender and history of prior COVID-19 infection was recorded. Another group of participants who experienced breakthrough infection (n=14) with SARS-CoV-2 within three months after receiving the primary vaccine doses were also included in this study. The demographic information of the study participants is shown in **Table 1**. All participants provided informed written consent. The study was approved by the Institutional Review Committee of the International Centre for Diarrhoeal Disease Research, Bangladesh (icddr,b), and the Institute of Epidemiology, Disease Control and Research (IEDCR).

**Table 1:** Demographic information of study participants.

	Vaccinees, n= 42	Patients, n=39	Healthy, n=20
Age, Median (range)	45.0 (26–79)	52.0 (22–68)	42.0 (22–63)
Sex, n (%)			
Male	25 (59.52)	22 (56.41)	12 (60.0)
Female	17 (40.48)	17 (43.59)	8 (40.0)
Primary vaccine doses, n (%)			
ChAdOx1 nCoV-19	28 (66.67)	20 (51.28)	
mRNA-1273	6 (14.29)	15 (38.46)	
BNT162b2	8 (19.05)	4 (10.26)	
Disease severity, n (%)			
Asymptomatic	–	10 (25.64)	–
Mild	–	9 (23.08)	–
Moderate	–	9 (23.08)	–
Severe	–	11 (28.21)	–

### 2.2 Specimens collection and processing

Blood samples were collected at different time intervals from COVID-19 patients and vaccinees for different immunological analyses. From patients, blood samples were obtained on the day of enrollment (day 1) and followed up on day 7, 28, 90, 180, 270, and 360 after infection. From the healthy participants, blood specimens were taken once [day 1 after confirmation of SARS- CoV-2 negativity by RT-PCR)]. From the vaccine recipients, blood specimens were collected on pre-first dose (Pre-V), one or two months following the first dose (Post dose 1), one month following the second dose (Post dose 2), and six months (6M) after the initial primary COVID-19 vaccination. Serum was separated from each blood specimen after centrifugation and stored at -70°C for further analyses.

### 2.3 Determination of RBD-specific antibodies

Enzyme-Linked Immunosorbent Assay (ELISA) was used to determine the RBD-specific IgG antibodies as described previously (7, 17). Briefly, 96 wells ELISA plates (Nunc^®^ MaxiSorp™, ThermoFisher) were coated with 1 μg/ml SARS-CoV-2 RBD antigen (Wuhan variant, gifts from A. Schmidt lab, Ragon Institute, Boston MA) and incubated for one hour at room temperature followed by blocking with 5% nonfat milk. Serum samples were heat-inactivated and added to the plates (serially 4-fold diluted samples in 5% Milk- 1X PBS 0.05% Tween). A specific monoclonal antibody to RBD of known concentration (Mab CR3022, gifts from A. Schmidt lab, Ragon Institute, Boston MA) was added to the plate, a 2-fold, 8 serial dilutions were performed and plates were incubated for an hour at 37°C. Subsequently, goat anti-human IgG peroxidase-conjugated secondary antibodies (Jackson ImmunoResearch) and ortho phenylenediamine (Sigma) in 0.1 M sodium citrate buffer (pH 4.5) with 30% hydrogen peroxide (Merck) was added to the plates. Reactions were allowed for 20 minutes and optical density (OD) was measured at dual wavelength (450 nm and 570 nm) in an ELISA Reader (Biotek). The concentration of RBD specific antibodies (ng/mL) was quantified using isotype-specific anti-RBD monoclonal antibodies.

To determine serum IgG1, IgG2 and IgG3 and IgG4 subclasses antibody responses, 96-well was coated with RBD, and then serum samples (3-fold, 8 serial dilution) were added to the plate. After that, plates were incubated for an hour at 37°C. Plates were then loaded with mouse anti-human Fc specific IgG1, IgG2, IgG3 and IgG4 that were conjugated with HRP (Southern Biotech) and incubated for another hour at room temperature (RT). Subsequently, ortho- phenylenediamine (OPD) in 0.1 M sodium citrate buffer (pH 4.5) and 30% hydrogen peroxide were added to the plate to start the reaction. After 20 minutes, 1 M H_2_SO_4_ (25 µL) was added to terminate the reactions. End point titers were calculated as the reciprocal interpolated dilutions of the samples at 492 nm, which were 0.4 above the background as previously described (18).

### 2.4 Determination of nucleocapsid-specific IgG antibody

SARS-CoV-2 nucleocapsid-specific IgG antibody was determined using an in-house ELISA assay. Briefly, nucleocapsid antigen (1 μg/mL) was coated onto 96-well Nunc MaxiSorp plates (Thermo Fisher Scientific, Waltham, MA, USA) and was allowed to incubate for one hour at RT. Plates were blocked with 5% nonfat milk in phosphate-buffered saline for 30 minutes. Diluted serum samples were added to plates and these were then incubated at 37°C for one hour. Anti-nucleocapsid monoclonal antibody (AssayGenie, Dublin, Ireland) of known concentration was added to the plate and two-fold serial dilutions were performed. Following incubation, plates were washed five times with PBST. Subsequently, goat anti-human IgG conjugated with horseradish peroxidase (Jackson ImmunoResearch, West Grove, PA, USA) was used and plates were then incubated at room temperature for 30 minutes. Bound secondary antibodies were detected using substrate OPD (Sigma, St Louis, MO, USA) in 0.1 M sodium citrate buffer (pH 4.5) and 30% H_2_O_2_. Plates were left in the dark for 15 minutes at room temperature. OD was measured at 450 nm in the Eon (Biotek, Winooski, VT, USA) ELISA Readers. A standard curve was plotted with the OD values obtained from monoclonal antibodies, concentration of the anti-nucleocapsid IgG antibodies samples (ng/mL) were quantified.

### 2.5 Statistical analysis

Antibody responses at different time points after infection and vaccination were analyzed in this study. To compare immune responses in patients, statistical analysis was performed between healthy controls and post infection day points using Mann-Whitney U test. For vaccine recipients, statistical analysis was performed between pre- and post-vaccination using paired t test. Graph Pad Prism (version 6.0) was used for generating plots and analyses.

## 3 Results

### 3.1 RBD IgG and its subclasses after COVID-19 diseases

**Figure 1:**
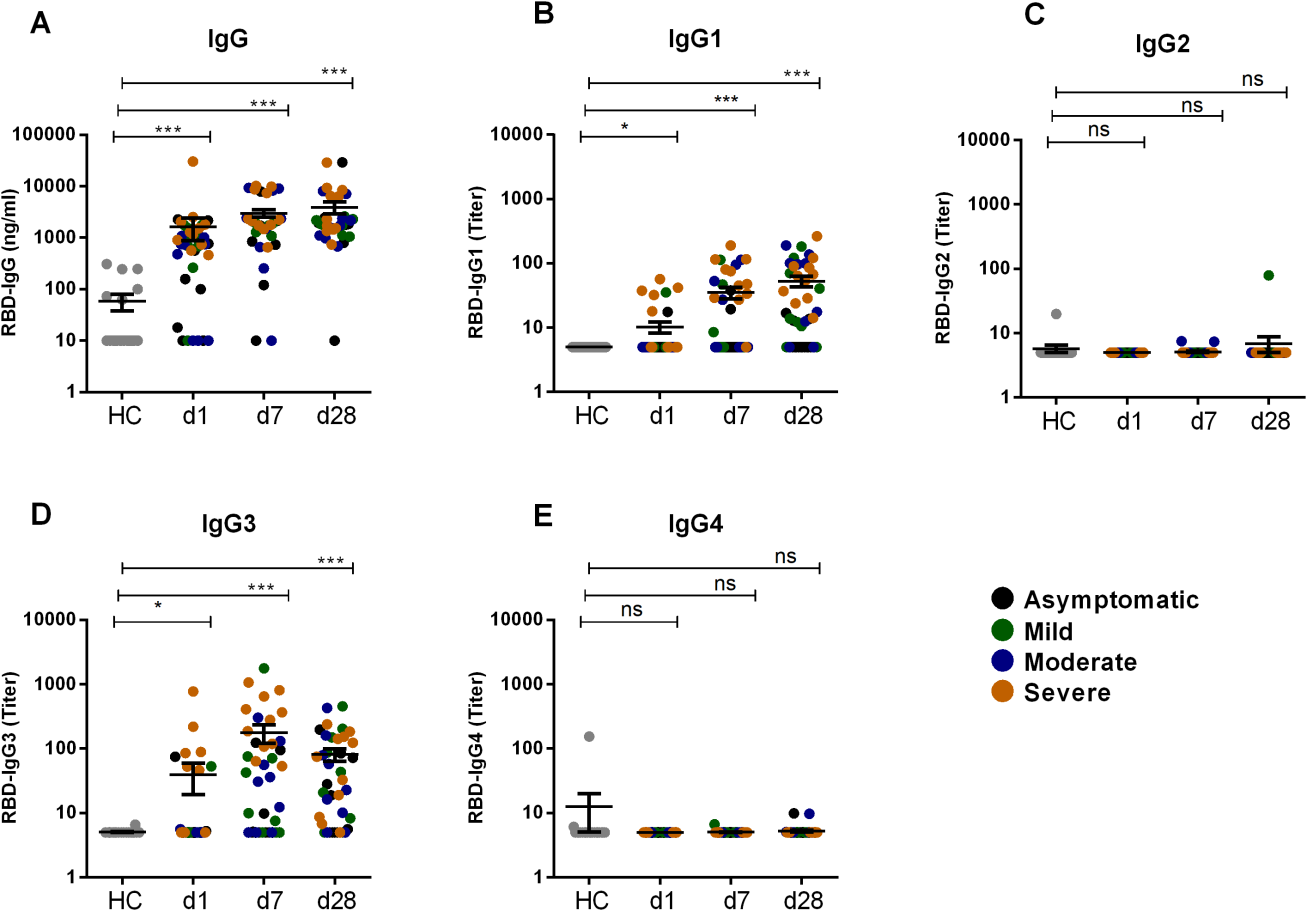
RBD specific IgG and its subclasses after SARS-CoV-2 natural infection. RBD specific IgG **(A)** and RBD specific IgG1 **(B)**, IgG2 **(C)**, IgG3 **(D)**, IgG4 **(E)** was analyzed in serum samples from naturally infected COVID-19 patients (n=39) and healthy controls (n=20). Samples were collected at different time points are indicated below the graph. Each symbol represents one individual where different color of dots indicates the category of patients as asymptomatic (black), mild (green), moderate (blue) and severe (orange). Statistical analysis was performed between healthy controls and patients using the Man-Whitney test. *P <0.05, ***P <0.0001, ns, not significant.

**Figure 2:**
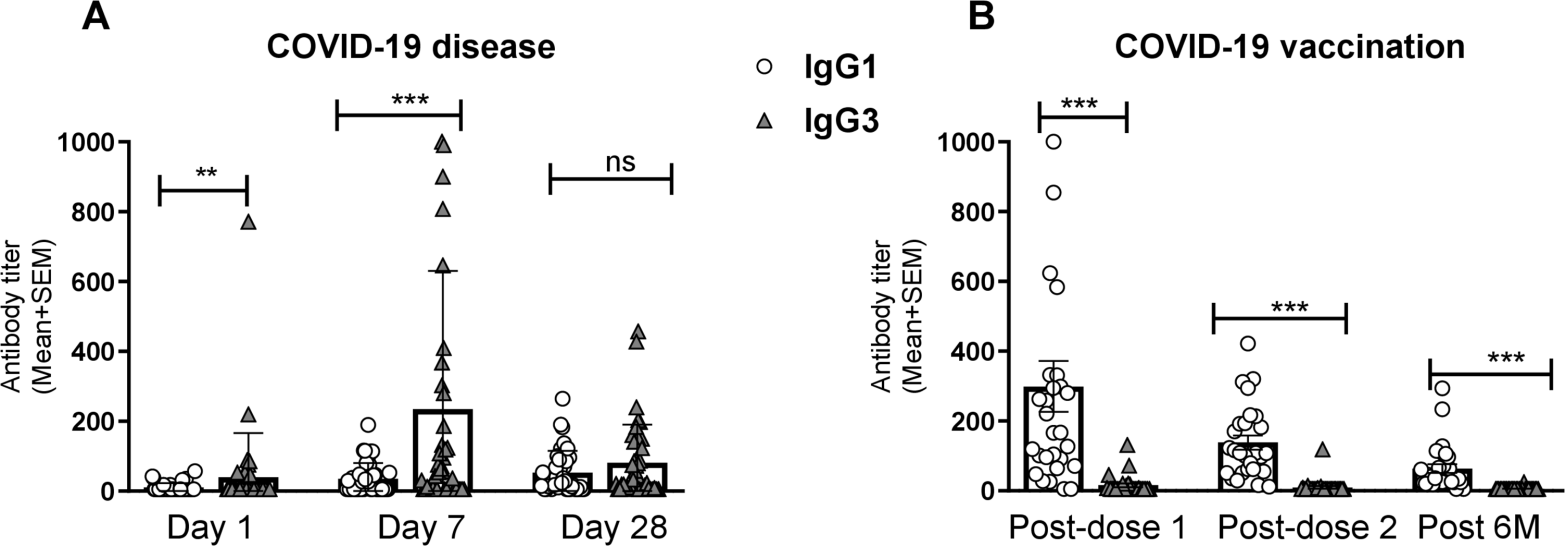
Comparison between RBD specific IgG1 and IgG3 after natural infection and vaccination. RBD-specific IgG1 and IgG3 antibody titers (mean +SEM) are shown in graph **(A)** after COVID-19 infection at Day-1, Day-7 and Day-28. RBD-specific IgG1 and IgG3 antibodies were compared at 1 month (post dose-1), 2/3 Months (post dose-2) and 6 months (Post 6M) after the first vaccination shown in graph **(B)**. Each dark bar represents mean of IgG3 with SEM whereas white bar represents IgG1. Statistical analyses were performed between IgG1 and IgG3 using the Wilcoxon test. **P <0.001, ***P <0.0001, ns, not significant.

We performed a comparative analysis of RBD-specific IgG and its subclass (IgG1, IgG2, IgG3, and IgG4) in COVID-19 patients with varying degree of COVID-19 disease severity, as well as in healthy controls (**Figure 1**). COVID-19 patients developed robust RBD-specific IgG antibody responses during acute phase of infection. Compared to healthy controls, patients exhibited significantly elevated levels (P<0.0001) of IgG antibodies on day 1, 7 and 28 following COVID-19 positivity (**Figure 1A**). Within the IgG subclasses, COVID-19 patients developed increased levels of IgG1 and IgG3 responses (**Figures 1B, D**). Additionally, we compared the level of IgG1 and IgG3 responses within patients across different time points after infection (**Figure 2**). IgG3 levels were found to be significantly higher than IgG1 on both day 1 (P<0.001) and day 7 (P<0.0001), indicating a dominance of IgG3 responses (**Figure 2A**). Conversely, no RBD specific IgG2 or IgG4 responses was observed in these patients.

### 3.2 RBD IgG and its subclasses after COVID-19 vaccination

**Figure 3:**
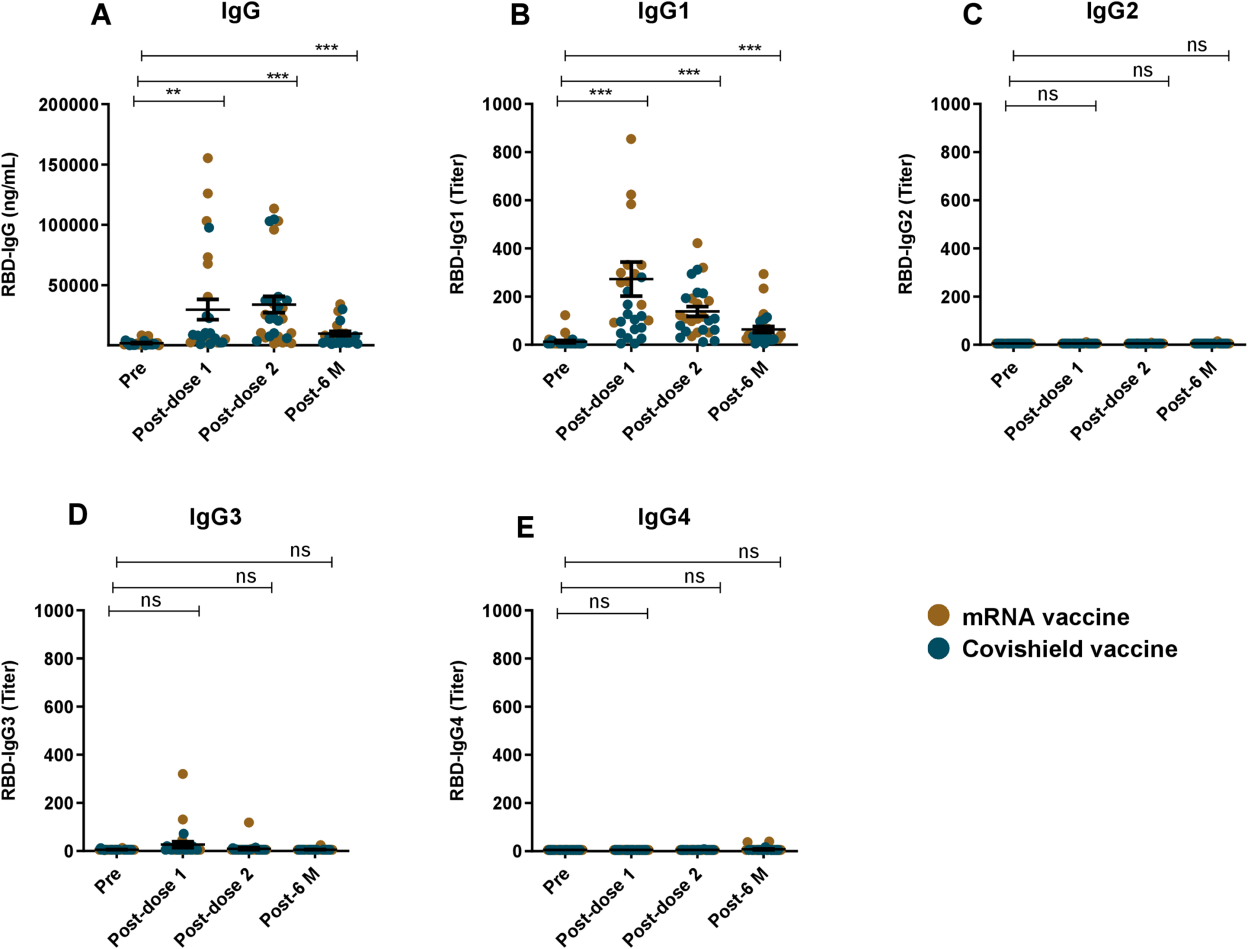
RBD specific IgG and its subclasses after COVID-19 vaccination. RBD specific IgG **(A)** and RBD specific IgG1 **(B)**, IgG2 **(C)**, IgG3 **(D)**, IgG4 **(E)** was analyzed in serum samples taken from COVID-19 vaccines (n=42). Samples were collected before vaccination (Pre), 1 month (post-dose 1), 2/3 Months (post-dose 2) and 6 months (post-6 M) after the first vaccination. Each symbol represents one participant where brown dots indicate mRNA and blue dots indicate Covishield, vaccine recipients. Statistical analysis was performed between pre and post-vaccination using the paired t test. **P <0.001, ***P <0.0001, ns, not significant.

Pre- and post-vaccination RBD-specific IgG and its subclass responses were evaluated in participants who received two primary doses of either Covishield or mRNA (Pfizer/Moderna) vaccines (**Figure 3**). COVID-19 vaccination induced robust IgG responses one month after both first and second doses. All vaccinees showed elevated level of serum IgG responses (P<0.0001) up to six months post-vaccination (**Figure 3A**). Among the IgG subclasses, a pronounced IgG1 response was observed following both doses of the Covishield and mRNA vaccines (**Figure 3B**), with IgG1 remaining dominant for up to six months. Interestingly, IgG3 responses were insignificant after both primary doses of COVID-19 vaccination (**Figure 3D**). This trend was more evident when IgG1 and IgG3 responses were compared (**Figure 2B**). IgG2 and IgG4 responses were nearly absent up to six months after primary vaccination (**Figure 2B, D**). These findings suggest that almost all RBD specific IgG responses generated by primary COVID-19 vaccinations were derived from IgG1 subclass.

### 3.4 RBD-IgG3 as a distinguishing immunological marker between infection and vaccination

**Figure 4:**
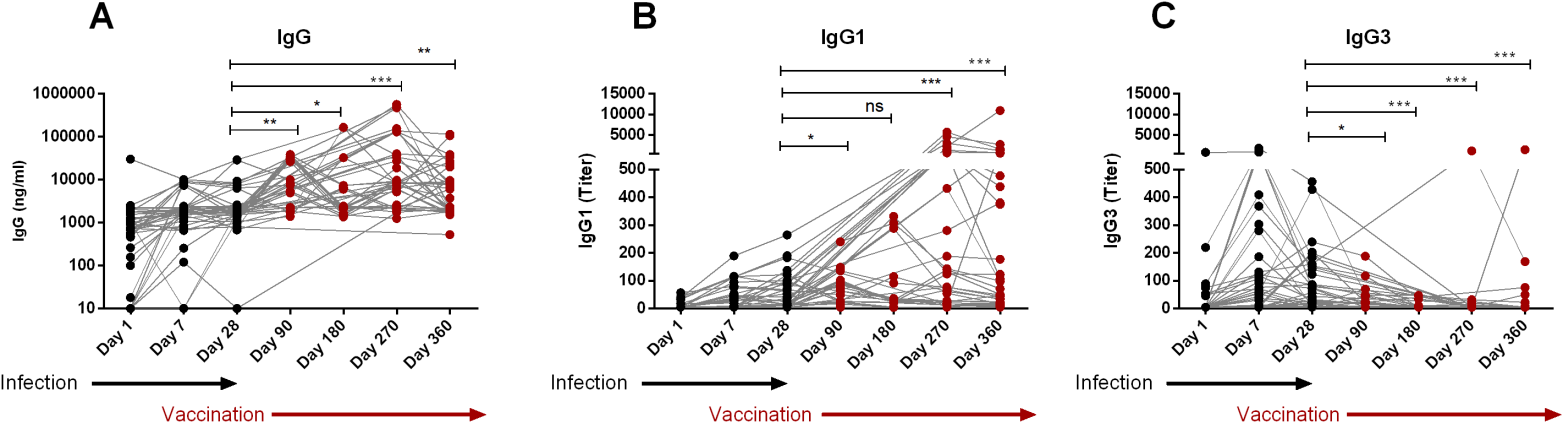
Boosting up of IgG1, not IgG3 after vaccination of naturally infected COVID-19 patients. RBD-specific IgG **(A)**, IgG1 **(B)** and IgG3 **(C)** were analyzed in serum samples from COVID-19 patients who received COVID-19 vaccines after 90 days post-infection (n=39). Samples were collected after infection and vaccination at different time interval are indicated below the graph. Each symbol represents one individual. The black dots indicate the time points before vaccination and the red dots indicates the time points after administration of vaccine. Statistical analyses were performed between prior vaccination (Day-28) and after vaccination using the Man-Whitney test. *P <0.05, **P <0.001, ***P <0.0001, ns, not significant.

All COVID-19 patients of this study eventually received COVID-19 vaccines (Covishield/Pfizer/Moderna) around 90 days after infection. After vaccination, we again evaluated their IgG, IgG1 and IgG3 responses on day 180, 270 and 360 to understand whether vaccination could further enhance serum RBD-specific IgG1 and IgG3 levels (**Figure 4**). As expected vaccination significantly boosted IgG on both day 270 (P<0.0001) and 360 (P<0.0001) (**Figure 4A**), the time when these participants completed two doses of the primary vaccine. Serum IgG1 also increased significantly after vaccination (**Figure 4B**). In contrast, serum IgG3 levels did not increase further after vaccination (**Figure 4C**), but rather decreased (P<0.0001) on day 180 and 270 and 360 compared to pre-vaccination levels (day 28 or day 90).

**Figure 5:**
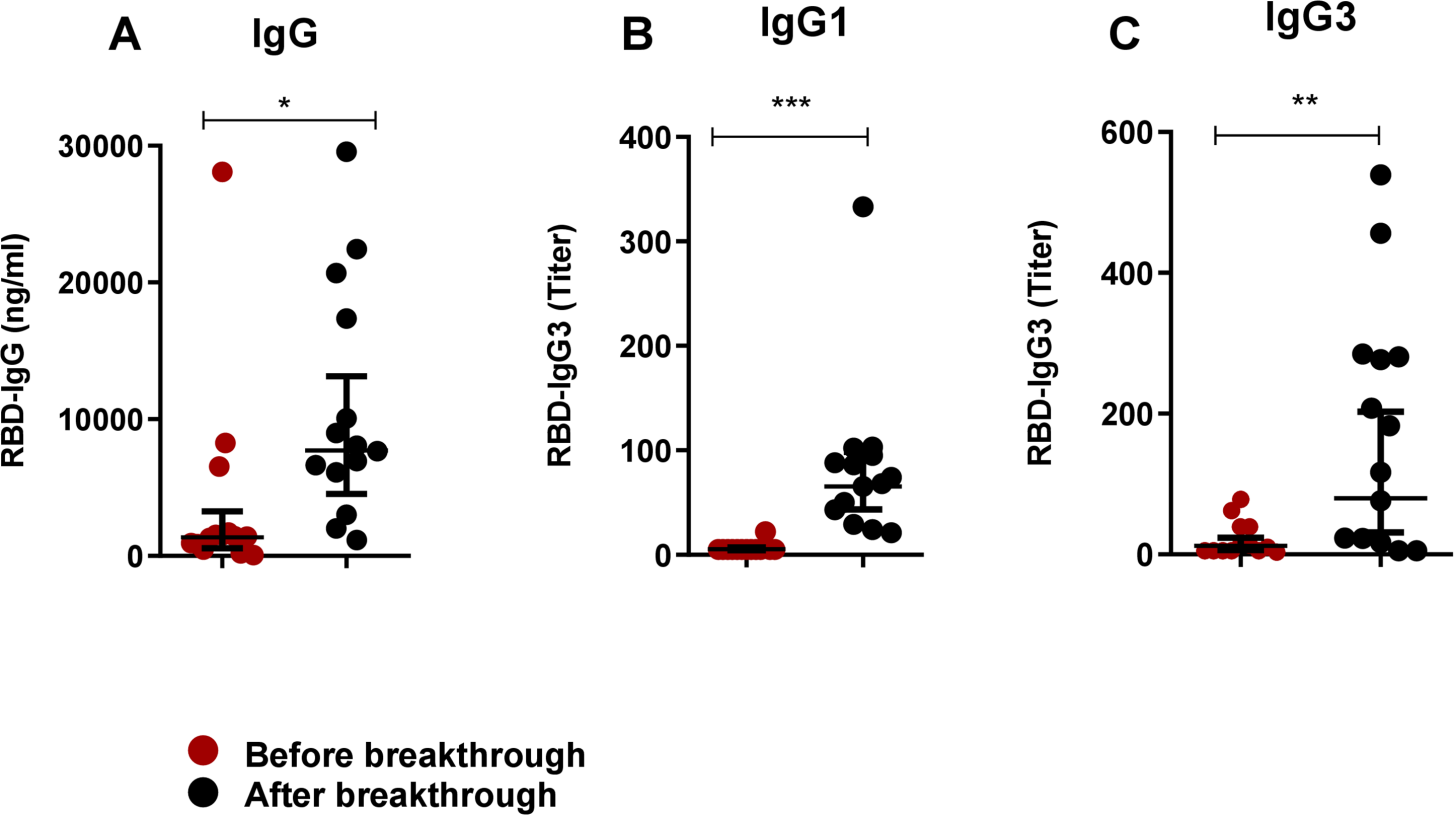
Boosting up of both IgG1 and IgG3 in breakthrough infection after primary vaccination. RBD-specific IgG **(A)**, IgG1 **(B)** and IgG3 **(C)** were analyzed in serum samples before (red dots) and after (black) breakthrough infection of COVID-19 vaccinated individuals (n=14). Each symbol represents one participant. Statistical analysis was performed between before and after breakthrough infection by using the Wilcoxon test. *P <0.05, **P <0.001, ***P <0.001.

We also evaluated antibodies in participants who experienced breakthrough infection after receiving two doses of the COVID-19 vaccine (**Figure 5**). Notably, prior to breakthrough infection, post-vaccination antibody levels of IgG, IgG1 and IgG3 were very low in these participants, which may not have been sufficient to prevent infection. Following breakthrough infection, we observed an increase in all studied antibodies; IgG (P<0.05, **Figure 5A**), IgG1 (P<0.0001, **Figure 5B**) and IgG3 (P<0.001, **Figure 5C**).

### 3.5 Nucleocapsid-specific IgG responses after COVID-19 diseases and vaccination

**Figure 6:**
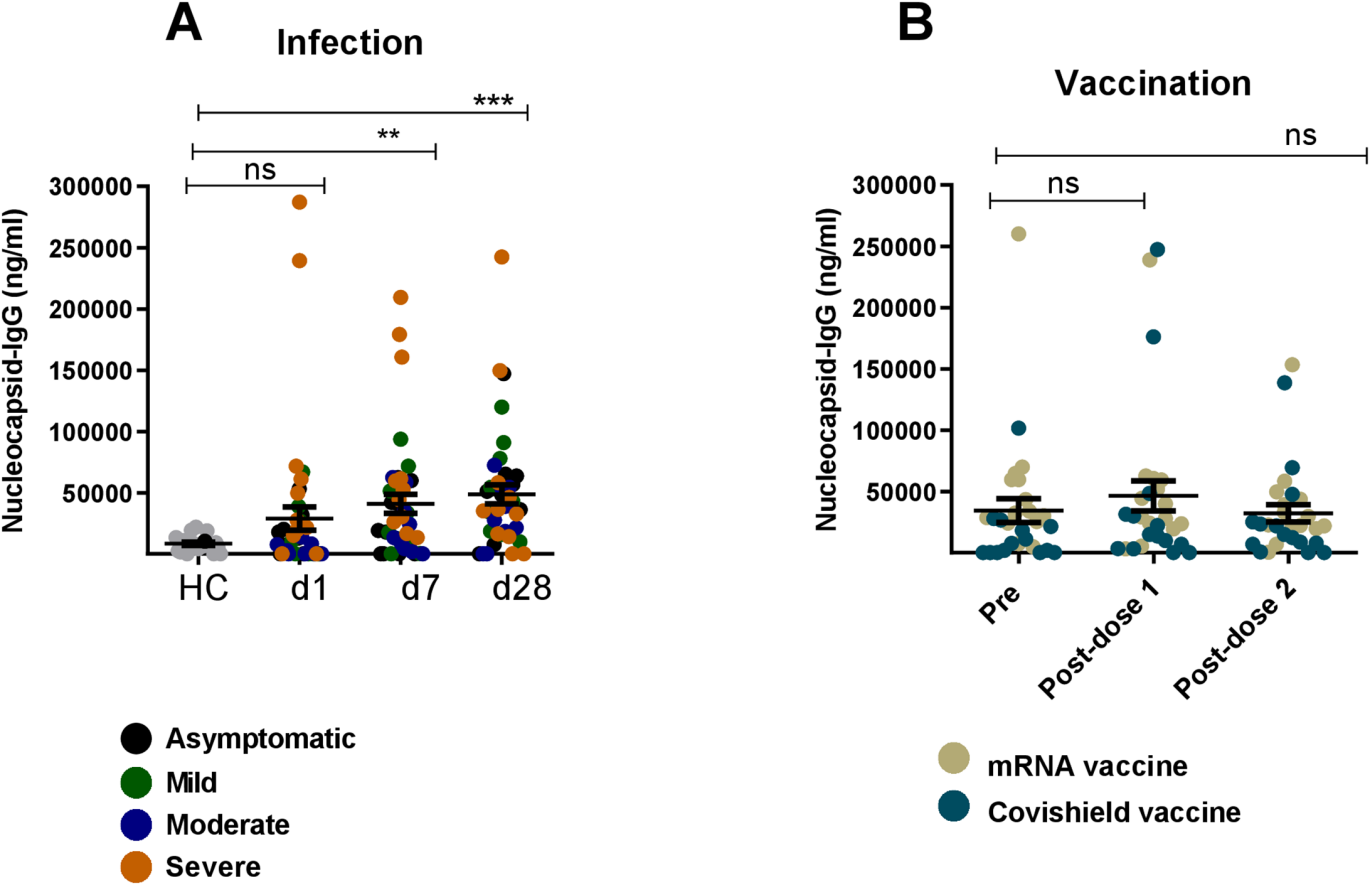
Nucleocapsid specific IgG responses after natural infection and vaccination. Nucleocapsid specific IgG antibody was measured in naturally infected COVID-19 patients (n=39) and healthy controls (n=20) **(A)** and COVID-19 vaccinated individuals (n=28) **(B)**. Samples were analyzed at day-1,7 and 28 after infection are indicated below graph **(A)** For vaccinated individuals, samples were analyzed before vaccination (pre) and after 1 month (post-dose 1) and 2/3 Months (post-dose 2) of vaccination indicated below graph **(B)** Each symbol represents one participant. In **(A)**, each dot represents one patient: asymptomatic (black), mild (green), moderate (blue) and severe (orange). In **(B)**, brown dots indicate mRNA and blue dots indicate Covishield, vaccine recipients. Statistical analysis was performed between healthy controls and infected patients using the Man-Whitney test and between pre and post vaccinated individuals using Wilcoxon test. **P <0.001, ***P <0.0001, ns, not significant..

Next, we evaluated SARS-CoV-2-specific nucleocapsid IgG responses in serum collected from COVID-19 patients and vaccinees. Patients had elevated levels of IgG responses against nucleocapsid antigen on day 1, 7 and 28 as compared to healthy controls (**Figure 6A**). As expected, serum antibodies from vaccinees did not respond to nucleocapsid antigens since this antigen is absent in both Covishield and mRNA-based vaccines (**Figure 6B**).

## 4 Discussion

The findings of this study provide insights into the humoral immune response to SARS-CoV-2, highlighting the distinct differences in the antibody profiles elicited by natural infection compared to vaccination. The most important finding of this study is the differential dynamics of serum spike RBD-specific IgG3 responses. Individuals naturally infected with SARS-CoV-2 exhibited elevated levels of both RBD-specific IgG1 and IgG3 antibodies; with IgG3 dominating in the early phase (day 7) of infection followed by a subsequent increase of both IgG3 and IgG1. Conversely, COVID-19 vaccination predominantly induced IgG1 responses without a rise in IgG3. This pattern was more evident in individuals who had previously been infected and then vaccinated, where a significant rise of IgG1, but not IgG3, was observed. However, following breakthrough infection in vaccinated participants, both IgG1 and IgG3 levels increased. These results -suggest that RBD-specific IgG3 only increased upon natural COVID-19 infection rather than vaccination. Additionally, other finding regarding nucleocapsid-specific IgG responses aligned with our expectations. Nucleocapsid specific serum IgG responses were present in COVID-19 patients but absent in vaccine recipients. Several studies investigating the nucleocapsid -specific humoral response reported a rapid decline in the antibody responses within a year post-infection that also depended on disease severity (19, 20). Overall, this distinction of RBD-IgG3 and nucleocapsid-IgG in patients versus vaccinees highlights their potential as serological markers for distinguishing vaccine-induced immunity versus immunity acquired through natural infection.

Serum antibodies, including IgG, exhibit multiple functions due to their polyclonal characteristics (21). Each of the human IgG subclass antibodies, IgG1, IgG2, IgG3, and IgG4, has unique characteristics and effector functions driven primarily by the Fc portion of the antibody molecule, including influencing complement activation and Fc receptor (FcR) binding. Antibodies against SARS-CoV-2 infection and vaccination is well studied (12, 17, 22). The elevated levels of RBD-specific IgG observed in COVID-19 patients during the acute phase of infection align with previous studies that have demonstrated a robust antibody response following SARS-CoV-2 infection. Our findings of increased SARS-CoV-2-specific IgG1 and IgG3 in patients is also in line with other studies showing the dominance of IgG1 and IgG3 antibodies after COVID-19 diseases, while IgG2 and IgG4 responses were minimal (16, 23). Studies have also shown that levels of IgG1 and IgG3 depend on disease severity, with higher levels of RBD-specific IgG1 and IgG3 observed in severe COVID-19 patients compared to those with milder disease (21, 24).

Our analysis further reveals a substantial difference in the levels of RBD specific IgG1 and IgG3 responses after infection. The predominance of IgG1 and IgG3 responses emphasizes their potential roles in the early immune defense against SARS-CoV-2. Serum RBD-specific IgG3 showed significantly higher levels than IgG1 during the early phase of infection (days 1 and 7), suggesting that IgG3 might play a critical role in mediating early antiviral activity, possibly due to its superior ability to fix complement and mediate antibody-dependent cellular cytotoxicity (ADCC) more effectively compared to other subclasses. This observation is supported by another study that showed a substantial difference in spike-specific IgG subclass composition, where a greater proportion of S1 and RBD-specific IgG3 was associated with COVID-19 severity in both a convalescent and acutely ill hospitalized patients (23). However, spike-specific IgG1, rather than IgG3, has been correlated with *in vitro* viral neutralization (23).

The robust IgG1 response observed following vaccination with both Covishield and mRNA vaccines indicates that these vaccines effectively stimulate an immune response that mirrors, to some extent, the IgG1-dominant response seen in natural infection. However, the lack of a significant IgG3 response post-vaccination is noteworthy (25, 18). This finding suggests that while vaccination induces a strong overall IgG response, the nature of the immune response may differ from that of natural infection, particularly in the context of IgG subclass distribution. The near absence of IgG2 and IgG4 responses post-primary vaccination is note-worthy, consistent with the response seen before in Bangladesh and other population (25, 16, 18). IgG4 responses have been shown to increase after booster COVID-19 mRNA vaccination (18, 25, 26). Another important observation was the lack of induction of IgG3 levels after vaccination, despite a concurrent increase in overall IgG and IgG1 levels, suggests that IgG3 may be more closely associated with the immune response to active viral replication rather than vaccination-induced immunity. This characteristic makes IgG3 a potential immunological marker for distinguishing between infection-induced and vaccine-induced immunity, which could be valuable in clinical and epidemiological settings.

The study also sheds light on the antibody response in individuals who experienced breakthrough infections after vaccination. The significant increase in IgG, IgG1, and IgG3 levels following breakthrough infection indicates that natural infection, even after vaccination, can further boost the immune response. This finding suggests that while vaccination provides robust protection, breakthrough infections may still occur in the absence of sufficient IgG, IgG1, or IgG3 levels. The ability of breakthrough infections to enhance these antibody responses highlights the potential for such infections to contribute to long-term immunity, possibly by promoting a more balanced and comprehensive immune response. Overall, this study provides important insights into the dynamics of IgG subclass responses following SARS-CoV-2 infection and vaccination. The dominance of IgG1 and IgG3 in natural infection, contrasted with the selective IgG1 response after vaccination, suggests that these subclasses play distinct roles in immunity to SARS-CoV-2. The unique behavior of IgG3, in particular, presents an opportunity to use this subclass as a marker to differentiate between infection and vaccination.

This study has some limitations including relatively small sample size because of the criteria to choose for subclass IgG and nucleocapsid analysis. Another limitation is, based on the interview question, that we considered that all the vaccinees were non-infected prior to vaccination but we can nor rule out the possibility of asymptomatic infection prior to vaccination. Despite these limitations, our findings have significant implications for understanding immune protection against COVID-19 and for guiding the development of future vaccines and therapeutic strategies. Further research is needed to examine the long-term implications of these IgG antibody responses and their relationship with clinical outcomes, including protection against reinfection and breakthrough infections.

## Data Availability

The raw data supporting the conclusions of this article will be made available by the authors, without undue reservation.
